# Herpesviridae Reactivation and Therapeutic Outcomes in Patients with Ulcerative Colitis Treated with Tofacitinib

**DOI:** 10.3390/jcm15187123

**Published:** 2026-09-14

**Authors:** Sivan Harnik, Naama Lang, Shiran Yehezkel, Ella Fudim, Miri Yavzori, Orit Piccard, Orel Finkel, Tal Meningher, Or Lya Abayev, Michal Tepperberg Oikawa, Sara Dovrat, Rachel Sihrazi, Shomron Ben-Horin, Bella Ungar

**Affiliations:** 1 Department of Gastroenterology, Sheba Medical Center Tel Hashomer, Ramat Gan 5265601, Israel; naamala14@gmail.com (N.L.); shiranye@gmail.com (S.Y.); miri.yavzori@sheba.health.gov.il (M.Y.); tal.meningher@sheba.health.gov.il (T.M.); liaor.abayev@sheba.health.gov.il (O.L.A.); shomron.benhorin@gmail.com (S.B.-H.); bella.geyshis.ungar@gmail.com (B.U.); 2 Gray School of Medicine, Tel-Aviv University, Tel Aviv 6997801, Israel; 3 Central Virology Laboratory, Sheba Medical Center, Ministry of Health, Ramat Gan 5265601, Israel; michal.teperberg@sheab.health.gov.il (M.T.O.); sara.dovrat@sheba.health.gov.il (S.D.); rachel.shiraze@sheba.health.gov.il (R.S.)

**Keywords:** ulcerative colitis, tofacitinib, *Herpesviridae*, HSV, VZV, Janus kinase inhibitor, viral reactivation, salivary PCR, fecal calprotectin

## Abstract

**Background:** Ulcerative colitis (UC) is a chronic inflammatory bowel disease. Historically, anti-tumor necrosis factor (TNFα) agents have been the mainstay of biological therapy. Recently, Janus kinase inhibitors (JAK-I) have emerged as oral alternatives. However, despite their acknowledged efficacy, concerns remain regarding viral reactivation, particularly *Herpesviridae* viruses. **Aims:** This study assesses the incidence of Herpesviridae (HSV and VZV) reactivation and the effect on the clinical course of UC patients treated with tofacitinib (JAK-I). **Methods:** This prospective cohort study monitored 27 UC patients starting treatment with tofacitinib for clinical and inflammatory disease activity using the simple clinical colitis activity index (SCCAI) and fecal calprotectin. Saliva samples from 24 patients were also analyzed by PCR for *Herpesviridae* reactivation. Follow-up duration for each patient was up to 42 months. The majority of samples (118/172) were collected over the first 12 months of therapy. **Results:** Clinical remission (SCCAI ≤ 3) was achieved in 48.1% of the patients. Reactivation of HSV and VZV was detected in 33.3% (8/24) and 4.2% (1/24) of tofacitinib-treated patients, respectively, with no symptomatic outbreaks, and in 4.2% (1/24) of patients primarily infected with HSV. Clinical disease activity was not associated with positive or negative HSV in saliva (66.7% vs. 33.3%, respectively, *p* = 0.21, R = 2.00, 95% CI 0.85–4.69); however, the study was not powered for this comparison. Similarly, the study was unable to detect or exclude an association between calprotectin levels in patients with or without *Herpesviridae* reactivations (*p* = 0.87). **Conclusions:** *Herpesviridae* virus (HSV/VZV) reactivation under tofacitinib treatment within the study cohort was asymptomatic, and the study was unable to detect or exclude an association with disease activity. No symptomatic herpes zoster reactivation occurred; however, the small cohort cannot exclude a clinically meaningful risk.

## 1. Introduction

Approximately 15–20% of patients with ulcerative colitis (UC) experience moderate–severe clinical symptoms, including diarrhea, rectal bleeding, and abdominal pain. Until recently, the mainstay treatment for patients with moderate–severe UC consisted of monoclonal antibodies that bind and inhibit tumor necrosis factor alpha (TNFα). This reduces the inflammatory stimulation caused by the cytokine, including induction of interleukin-6, promotion of leukocyte migration, and activation of neutrophils [1].

Janus kinases (JAKs) are intracellular enzymes that phosphorylate, and thereby activate, signal transducers and activators of transcription (STATs) that influence hematopoiesis as well as immune function [2]. There are several types of JAK enzymes, all of which require two JAK molecules for their action. Notably, JAK activation is crucial for an immune response against viral pathogens, and some viruses employ special techniques to avoid such activation [3]. For example, Herpes Simplex Virus 1 (HSV-1) uses a ubiquitin-specific protease, UL36USP, to block the activation of JAK1 and thus suppress interferon signaling [4]. Varicella zoster virus (VZV), another member of the *Herpesviridae* family, which causes herpes zoster in adults, also inhibits the JAK-STAT pathway [5].

Recently developed small molecule inhibitors of JAKs (JAK-I) have now been shown to induce clinical and endoscopic disease remission in UC [6]. Tofacitinib is an orally administered JAK-I that inhibits almost all combinations of JAK, albeit with different potencies [7]. However, treatment with these agents has been associated with a three-fold increase in VZV reactivation compared to the general population [8]. Although there is no clear relation to treatment duration, there is a positive relationship between tofacitinib dose and increased risk for VZV reactivation [9]. In addition, tofacitinib mildly increases the risk for HSV-1 infection in comparison to placebo, although it remains unclear whether the cases noted were primary infections or reactivation of the virus in an already infected individual [10]. There is also little information about the effect of a primary herpes infection/reactivation on the clinical course of UC, and specifically about whether asymptomatic reactivation can cause clinical deterioration in otherwise stable UC patients [11]. The current study was therefore designed to study the effect of tofacitinib therapy on *Herpesviridae* virus activation and the efficacy in treating UC.

## 2. Methods

### 2.1. Patient Population

Thirty UC patients were started on tofacitinib treatment during the years 2021–2025 in a single-center exploratory cohort study with no pre-specified confirmatory hypothesis. All patients were offered to join the trial; however, three declined. Therefore, 27/30 patients starting tofacitinib for the treatment of UC were followed prospectively. All patients followed the standard tofacitinib induction protocol of 10 mg of Tofacitinib twice daily for 8 weeks, followed by a maintenance dose of 5 mg twice daily.

Serum samples obtained prior to starting treatment were analyzed for VZV-IgG and HSV-IgG. Similarly, baseline stool samples were analyzed for calprotectin levels, and new stool samples were collected serially every 3 months during follow-up. None of the patients were vaccinated against VZV prior to and during the study. Additionally, none of the patients received any antiviral prophylaxis during the study.

Saliva samples were collected monthly to monitor HSV and VZV infection using real-time PCR, and patients were interviewed every 30 days to assess the simple clinical colitis activity index (SCCAI).

Salivary HSV and VZV positivity served as the primary outcome. Secondary outcomes included episodes of loss of response/need for change in therapy/hospitalizations.

The study was approved by the ethics committee and all patients signed an informed consent form.

### 2.2. Clinical Scores—SCCAI

The index involves an evaluation of day/night bowel frequency, urgency of defecation, blood in stool, general health, and extra-colonic manifestations of UC. The SCCAI scores were calculated prospectively, and patients with SCCAI ≤ 3 were considered in clinical remission [12].

### 2.3. Sample Collection

Saliva samples—patients were asked to spit through a designated straw into a 2 mL collection tube until the amount of liquid saliva reached the 1 mL line. The straw was then discarded and the collection tube was closed tightly with a sterile cap and stored at −80 °C.

All saliva samples were handled at the Central Virology Laboratory, which is affiliated with the Ministry of Health and is located in the Sheba Medical Center.

### 2.4. Viral Serology and PCR Analysis

DNA extraction: Viral genomic DNA was extracted from 200 μL saliva using the magLEAD 12gC benchtop extraction machine (Precision System Science Co, Ltd. Matsudo-shi, Chiba, Japan), which utilizes automated magnetic bead technology. Remaining samples were discarded after the final PCR results were obtained. All samples were stored at −80 °C until analysis.

Viral HSV1, HSV2, and VZV DNA was detected by real-time PCR using TaqMan master mix (Eurogentec RT-QP2X03-50) with readout by the ABI 7500 instrument (Applied Biosystems, Foster City, CA, USA). Briefly, each reaction was prepared in a total volume of 25 μL, using the TaqMan master mix (Eurogentec RT-QP2X03-50), TaqMan primers (300 nM per reaction), labeled probe (200 nM), and 10 μL of DNA extract [13]. real-time PCR was performed under the following conditions: 2 min at 50 °C, 10 min at 95 °C, 50 cycles of 15 s at 95 °C, and 1 min at 60 °C. Primers and probes for the detection of each virus were used as follow: VZV-F: 5′tacacgtgatactgagacaaagcg 3′, VZV-R: 5′ tggtgttggacgcggtg 3′, VZV-probe 5′ tccatccctgggcc 3′, HSV1-F: 5′ ggcctggctatccggaga 3′, HSV1-R: 5′ gcgcagagacatcgcga 3′, HSV1-P: 5′ cagcacacgacttggcgttctgtgt 3′, HSV2-F: 5′ agatatcctctttatcatcagcacca 3′, HSV2-R: 5′ ttgtgctgccaaggcga 3′, HSV2-P: 5′ cggcggcgttcgtttgtctg 3′. Limit of detection for each virus is as follows: VZV 16 copies per reaction (800 copies per mL), HSV1 14 copies per reaction (700 copies per mL), HSV2 17 copies per reaction (850 copies per mL). RNase P served as an internal control for each sample. The absence of RNase P detection indicated the presence of PCR inhibitors. Whenever the Ct value was weakly positive (above Ct 37), the test was repeated. If the values in the repeated test were between 37 and 40, the result was reported as “weakly positive”. Each test included a positive control for each virus at a predetermined Ct value. Whenever the Ct value of any of these controls deviated by more than one standard deviation from the expected value, the test was considered invalid and was canceled.

Detection of HSV-IgG antibodies: Serological testing for HSV (either 1 or 2) specific IgG antibodies used the enzyme-linked immunosorbent assay (ELISA) commercial kit for HSV [SERION ELISA classic HSV1/2 IgG kit (Institute Virion\Serion GmbH, Wurzburg, Germany)] according to the manufacturer’s instructions.

Kit sensitivity: 95.5%; specificity: >99%. Results > 30 U/mL were considered positive, values 20–30 equivocal, and values < 20 U/mL were considered negative.

Detection of VZV-IgG antibodies: VZV IgG antibodies were detected with the Vidas^®^ Assay (Biomerieux VIDAS, Lyon, France), which combines a two-step sandwich enzyme immunoassay method with a final fluorescence detection (ELFA). Results ≥ 0.90 U were considered positive, while results below the negative threshold (0.60 U) were considered undetectable. A test with results between 0.6 and 0.9 U was repeated.

### 2.5. Stool Calprotectin

Calprotectin was assessed using a home stool smartphone-based kit (CalproSmart, Lyskar, Norway). In case of exacerbations, calprotectin was measured at the hospital using a commercially available ELISA (Quantum Blue Calprotectin Quantitative Lateral Flow Assay, LF-CAL 125, Buhlmann Laboratories, Switzerland) [14,15].

### 2.6. Statistical Analysis

Continuous variables were expressed as the median and interquartile range (IQR). All reported *p* values were two-sided, and a *p* value < 0.05 was considered statistically significant. All *p* values are unadjusted. Mann–Whitney tests were used to compare continuous variables, and Fisher’s exact test was used when comparing categorical data (MedCalc Software version 12.2.1.0, Mariakerke, Belgium).

## 3. Results

### 3.1. Tofacitinib and VZV/HSV Infection

**Figure 1:**
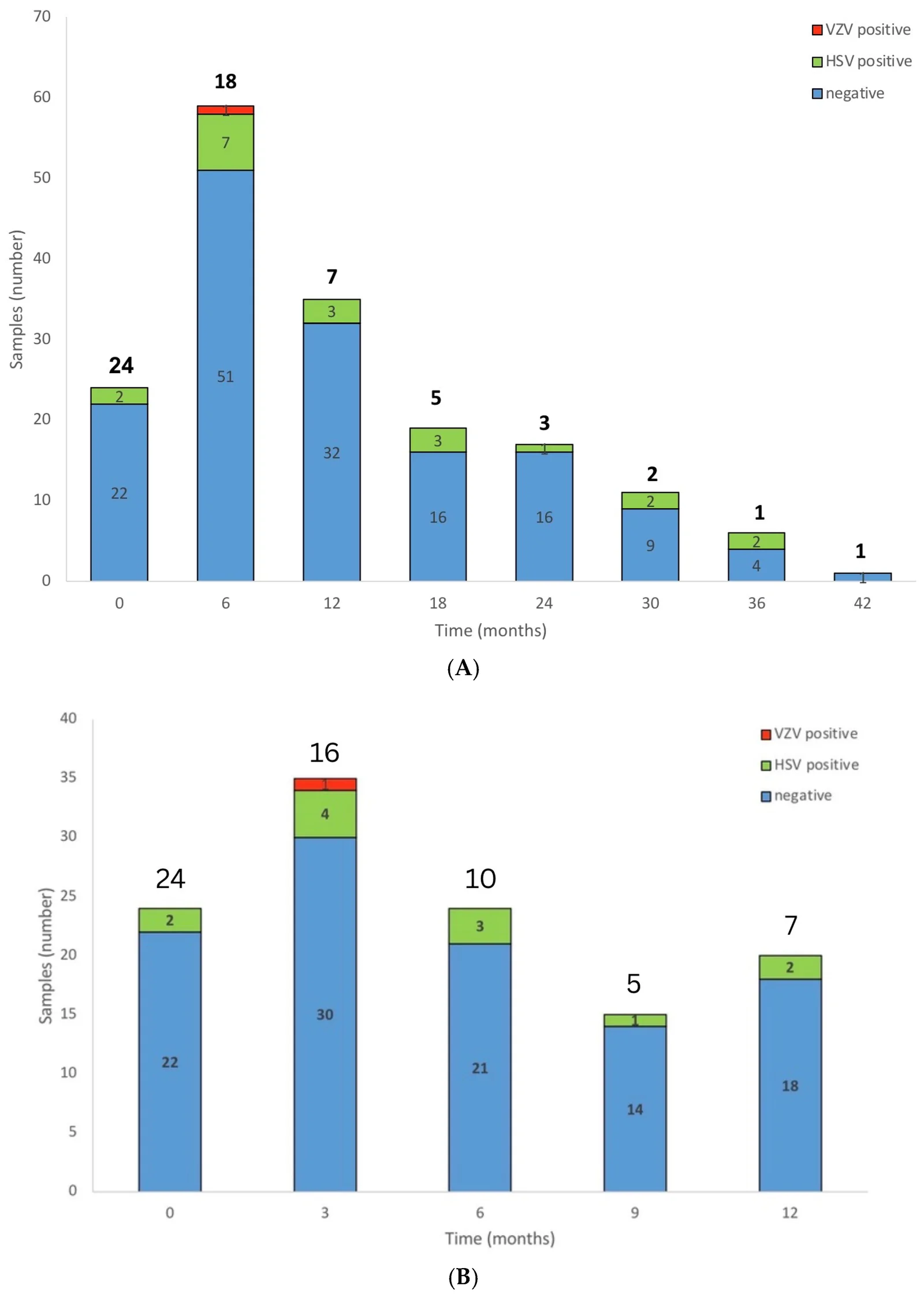
(**A**) HSV and VZV infection detection in saliva samples throughout the study period is shown. Patients provided periodic monthly saliva samples for up to 42 months throughout the 46 total months of data collection (first patient enrollment—last patient follow-up). The intervals are labeled by their upper limit. While most samples were negative, positive HSV saliva samples were detected throughout the study, with the only positive VZV saliva sample detected at 6 months from the start of the study. The Y axis is the number of samples provided in each period, with the X axis indicating time from the start of the therapy for each saliva sample. The number above each column indicates the number of different patients who provided samples during the 6-month intervals. (**B**) HSV and VZV infection detection in saliva within the first 12 months of therapy, 68% (118/172) of total saliva samples were obtained during the first 12 months,; the intervals are labeled by their upper limit. The y-axis indicates the number of samples collected over each 3-month period, with the number above each column indicating the number of different patients who provided t he samples during each time period.

Out of the patients with prospectively available saliva samples (24/27 patients), 87.5% (21/24) of patients were VZV IgG positive prior to initiation of tofacitinib treatment, and 62.5% (15/24) of patients were HSV IgG positive. In total, 172 saliva samples were collected from 24 patients over 46 months, with periodic sampling of each patient over up to 42 months of follow-up to detect HSV1, HSV2, and VZV infections. The results revealed that 11.6% (20/172) of all saliva samples were positive for HSV, with only one sample positive for VZV (Figure 1).

The results of per-patient analysis detected 9 (33.3% (95% CI 18.8–59.4%) cases of positive HSV infections (88.9% HSV-1 and 11.1% HSV-2) by PCR of saliva samples. In fact, 2 out of the 9 patients were already HSV-1 positive at time point 0. Of the nine patients, 8/9 (88.9%) already had positive serology for HSV-IgG prior to therapy initiation, indicating viral reactivations. One patient (11.1%) presented with an emergent HSV-1 infection (negative HSV-IgG serology at initiation). All cases were clinically asymptomatic for HSV infection. Most of the positive HSV saliva samples were identified within 14 weeks of tofacitinib commencement. There was an underpowered comparison in the clinical remission rate achieved by patients positive or negative for saliva HSV, with values of 66.7% (6/9) and 33.3% (5/15), respectively (*p* = 0.21, R = 2.00, 95% CI 0.85–4.69). Similarly, the study was unable to detect or exclude an association in calprotectin levels between patients who were HSV positive versus HSV negative upon initiation of treatment (*p* = 0.87; both groups with median = 1000 µg/g).

VZV positive infection was documented only once (1/24) during the monthly study follow up, at 8 weeks of therapy (4.2% (CI 95% CI 0.1–21.1%), in a patient who was also positive for VZV IgG at the beginning of treatment. This patient did not achieve clinical remission and discontinued tofacitinib treatment 10 weeks into the study.

### 3.2. Clinical Outcomes of the Prospective Tofacitinib Cohort

Approximately half (44.4%, 12/27) of the tofacitinib-treated patients were female with a median age of 36 years. Similarly, 51.9% (14/27) of the patients were concomitantly treated with steroids when commencing tofacitinib (Table 1). The majority (81.5%, 22/27) of the patients had received biological therapy prior to tofacitinib, with 55.6% (15/27) suffering from extensive pancolitis.

**Figure 2:**
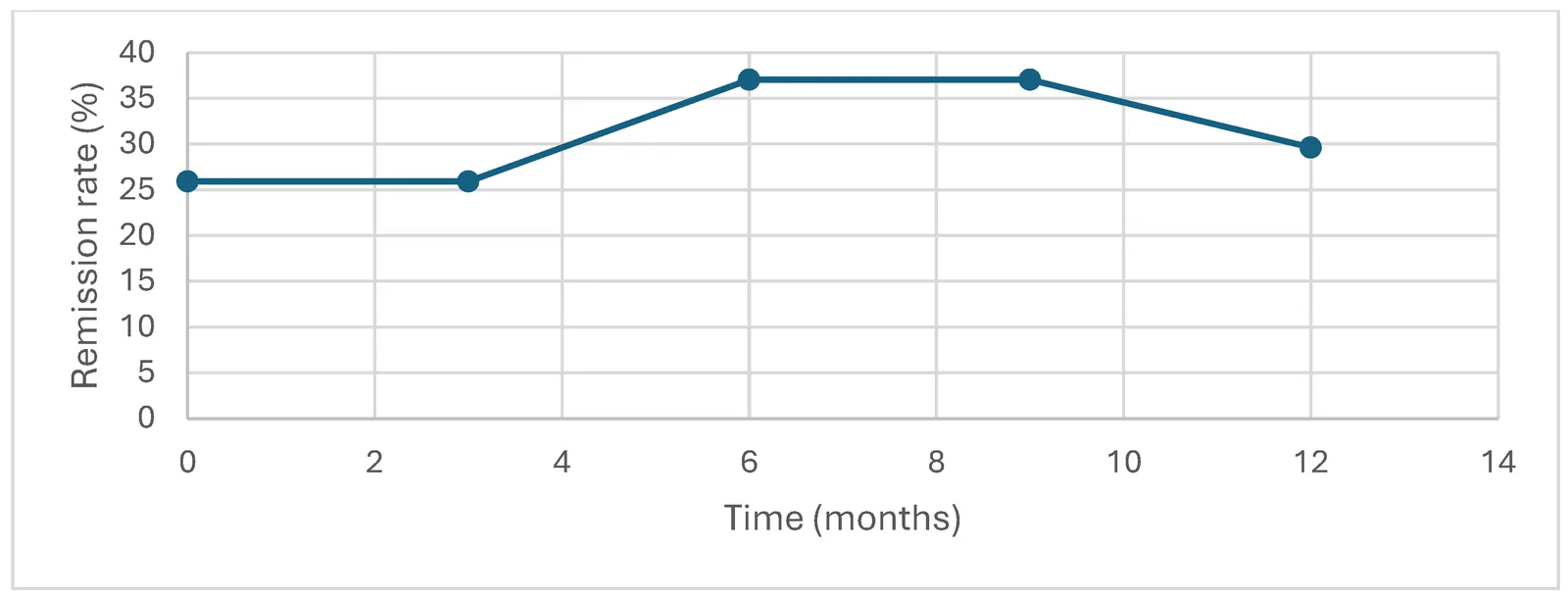
Clinical remission rate over time: Percentage of patients in clinical remission out of the total study cohort over time in the tofacitinib cohort.

Overall, 48.1% (13/27) of patients achieved clinical remission when treated with tofacitinib. Average and median times to remission were 17.9 and 13 weeks, respectively (Figure 2). Notably, 20 patients had clinical disease activity based on their SCCAI scores at study commencement. Only 3 of them (15%) reached clinical remission by the end of the study period. Furthermore, 4/13 (30.8%) of patients achieving clinical remission on tofacitinib therapy lost clinical response over the course of the study (with a median time of 32.4 weeks), and 66.6% of the total cohort (18/27) stopped therapy due to loss of response before 12 months (median follow-up 4 months, IQR 1–9.5 months). No patients were lost to follow-up. A total of 29.6% of tofacitinib-treated patients maintained remission at the end of the 12-month follow-up (Figure 2). Over the course of the study, 7.4% (2/27) of the patients were hospitalized and subsequently switched therapy.

### 3.3. Biomarker Remission

**Figure 3:**
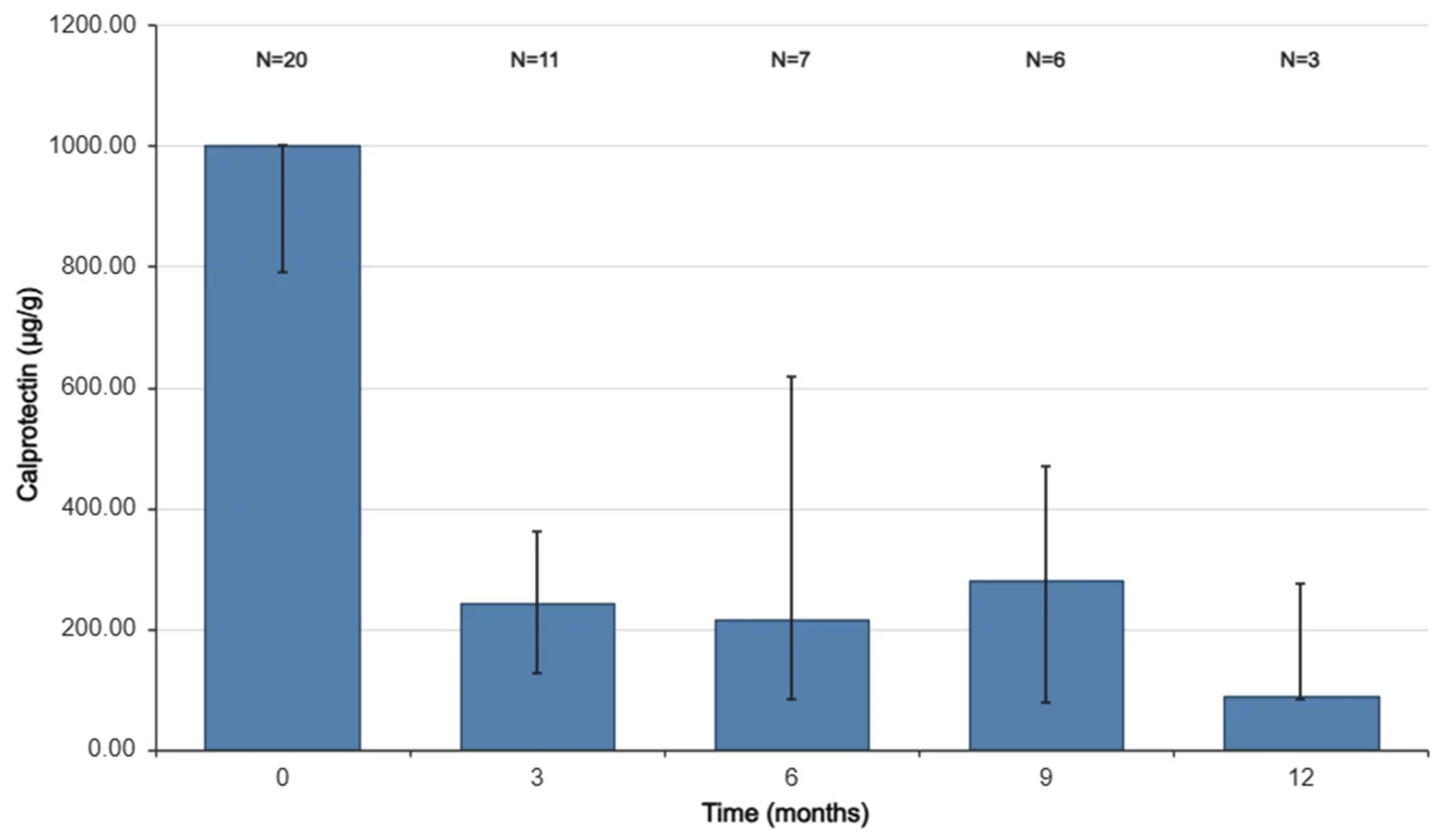
Median calprotectin levels throughout the study period, median calprotectin levels at months 0, 3, 6, 9, and 12 for the tofacitinib cohort; each month has interquartile 1 and 3 marked within the error bars and the number of patients marked by n = x above the bar.

Median baseline calprotectin levels were 1000 µg/g at the start of the study and decreased to a median of 87 µg/g in patients achieving remission and continuing therapy up to 12 months (Figure 3). Most (75%, 6/8) of the patients achieving clinical remission exhibited calprotectin levels of below 100 µg/g, with a median of 73.5 µg/g, which is defined as normalization. In comparison, none (0/12) of the patients who did not achieve clinical remission achieved normalization of calprotectin levels, with a median of 930 µg/g. This difference is statistically significant (*p* = 0.001). It should be noted that the number of patients contributing calprotectin samples declined throughout the trial (N = 20, 11, 7, 6, and 3 at months 0, 3, 6, 9, and 12, respectively), with the measurement of 12 months containing only three patients. As patients discontinuing tofacitinib for non-response were censored, those remaining at later timepoints are progressively enriched for treatment responders. Therefore the trajectory in Figure 3 should not be interpreted as a treatment effect.

## 4. Discussion

HSV and VZV infections are prevalent throughout the world. The prevalence of HSV-1 in adults is 47.8% in the US and 67.4% in Europe, while the prevalence of HSV-2 in the western world is roughly 12% [16,17,18,19]. In our study, 62.5% of participants exhibited positive serology for HSV (referred to as a composite of HSV-1 and HSV-2), which is similar to the composite values in the US. In contrast to the chronic nature of HSV, characterized by painful oral or genital ulcers, a primary infection with VZV is associated with a transient rash and fever. Reactivation of VZV is termed “shingles” and is characterized by a dermatological rash accompanied by neuropathic pain. Almost 99% of the Western world population is exposed to VZV during their lifetime, and despite the increasing use of the shingles vaccine, which has reduced the prevalence of VZV reactivation, currently 23.8–30% of the European/US population are likely to suffer from the condition [20,21,22]. Our finding of one case of VZV reactivation (4.2% of patients), which is slightly lower than literature reports of 5.6% for tofacitinib-treated UC patients, may be explained by the small cohort size and 5 mg twice-daily maintenance dose [8].

Reactivations of VZV and other *Herpesviridae* virus infections in immunocompromised patients, and specifically in tofacitinib-treated patients, may be severe and atypical, extending beyond the dermis and causing potentially pre-malignant pathologies [23]. This could be related to the mechanism of action by which *Herpesviridae* exploit the inhibition of the JAK STAT pathway by the drug [24]. In our study, 53.3% (8/15) of the patients treated with tofacitinib who were known to be infected with HSV (based on serology) exhibited HSV reactivation in their saliva; two of those patients were already HSV positive on saliva PCR before tofacitinib commencement. However, this activation did not manifest clinically, nor did it appear to be correlated with UC severity. Our results are in accordance with previous reports that subclinical reactivations of HSV can be detected at much higher rates at the molecular level in oral and genital swabs taken from immunocompetent adults than the frequency of clinical reactivations. One American study reported that only 6% of swab-identified oral activations resulted in symptomatic lesions [25]. We can conclude that although tofacitinib-treated patients may be immunocompromised to some degree, the effect on the immune system is probably not as extensive as seen in severely immunocompromised organ-transplant patients. This also agrees with our findings (Figure 1) that tofacitinib users experience HSV reactivations at similar rates to the immunocompetent population, lower than that of severely immunocompromised organ transplant patients, where asymptomatic viral shedding can be as high as 80% [26].

Similarly to previous studies, the results of our prospective study demonstrate that tofacitinib may be used as an effective treatment option for UC. Clinical response to tofacitinib was achieved by a median of 13 weeks, which accords with recent clinical publications [27,28]. However, 14.8% (4/27) of the patients lost response 12–48 weeks from therapy commencement. By 12 months of therapy, only 29.6% (8/27) of patients were in clinical remission. This is probably related to the fact that most of the cohort had previously suffered from pancolitis (55.6%), were bio-experienced (81.5%), and were receiving steroids at the beginning of the follow-up period (51.9%). Interestingly, calprotectin values normalized in 75% (6/8) of clinical responders and in none (0/12) of the non-responders, demonstrating the association of calprotectin values with clinical indices in colonic, compared to ileal IBD [29].

Regarding study limitations, we must first address the small sample size of the study, which has rendered the sample size too small to statistically demonstrate or exclude an association between herpes reactivation and clinical remission. We estimate that to detect the observed difference in clinical remission between HSV-positive and negative patients (66.7% versus 33.3%) at 80% power and α= 0.05, two-sided with the same 9:15 allocation, requires approximately 72 patients, roughly three times the current cohort. However, as this was a ‘real life’ study, power was not calculated before initiation and all available patients who consented were included. Despite the total of only 27 patients, this was a prospective study of a unique and homogenous cohort—UC patients treated with tofacitinib, closely monitored both for Herpes viruses, clinical outcome, and biomarkers. Secondly, the ELISA analysis of HSV serology did not differentiate between HSV-1 and HSV-2. The HSV subtypes differ in pathogenesis, with HSV-1 primarily infecting the trigeminal ganglia, while HSV-2 tends to be more extensive throughout the body, including the genitalia, and is more likely to cause viremia [30]. Notably, however, none of the HSV-positive patients in our cohort exhibited any clinical manifestations of infection. Furthermore, unlike the baseline serology, the PCR analysis performed throughout the study for viral detection in all patients did differentiate between HSV1 and HSV2. Lastly, remission was assessed using the clinical SCCAI score and stool biomarker (calprotectin), without endoscopic evaluation.

To conclude, JAK inhibitors in general, and tofacitinib specifically, are common treatment choices for patients with UC, although often not the first line. The results within our small cohort have found that almost 50% of patients treated with tofacitinib and who remained on treatment reached clinical and biological remission at some point, even though 14.8% (4/27) experienced a flare-up throughout therapy and 66.7% (18/27) stopped therapy before 12 months. Another interesting finding is that despite inhibition of the JAK-STAT pathway, HSV reactivation was not more prevalent among UC patients treated with tofacitinib in the study than in the general population. Importantly, our study found no correlation between reactivation of HSV and disease severity as evidenced by calprotectin levels and clinical scores. Rates of VZV infections were also very limited among patients treated with tofacitinib within our small cohort. Our results are in accordance with previous reports of a low risk for VZV reactivation in response to JAK-I, especially in the era of inactivated anti-shingles vaccines. Larger corroborating studies are required in order to fully compare outcomes of therapy and to evaluate symptomatic and asymptomatic HSV and VZV reactivations in such patients.

## Figures and Tables

**Table 1:** Clinical and demographic parameters of the study cohort.

	Tofacitinib Cohort (N = 27)	
	**IQR ^†^**	**Median**
Age at induction (years)	26.5–48.5	36
Disease duration (years)	6–16	10
Age at diagnosis (years)	17–30	20
BMI ^‡^ at induction (kg/m^2^)	23.5–27.5	26.1
Baseline calprotectin level	791–1000	1000
Baseline SCCAI ^§^ score	2.5–9.5	8
	**N**	**Percent**
Female gender	12	44.4
Disease location (proctitis)	0	0
Disease location (left-sided colitis)	12	44.4
Disease location (pancolitis)	15	55.6
Concomitant steroid therapy	14	51.9
Concomitant immunomodulator therapy	0	0
Comorbidities	5	18.5
Prior immunomodulator therapy	10	37.0
Prior biological therapy	22	81.5
Number of previously trialed biological drugs		
Biologically naive (0 previous biological drugs)	5	18.5
1 previous biological drug	10	37
2 previous biological drugs	8	29.6
3 or more previous biological drugs	4	14.8
Prior vedolizumab therapy	16	59.2
Prior infliximab therapy	15	55.6
Smoking at induction	1	3.7

^†^—IQR—interquartile range ^‡^—BMI—body mass index. ^§^—SCCAI—simple clinical colitis activity index. All percentages use a denominator of 27 for the 27 patients in the study.

## Data Availability

The data presented in this study are available on request from the corresponding author due to patient privacy.
